# The Association Between Smoking and Electronic Cigarette Use in a Cohort of Young People

**DOI:** 10.1016/j.jadohealth.2017.11.301

**Published:** 2018-05

**Authors:** Katherine East, Sara C. Hitchman, Ioannis Bakolis, Sarah Williams, Hazel Cheeseman, Deborah Arnott, Ann McNeill

**Affiliations:** a Addictions Department, Institute of Psychiatry, Psychology and Neuroscience, King's College London, London, UK; b UK Centre for Tobacco and Alcohol Studies, Clinical Sciences Building, University of Nottingham, Nottingham, UK; c Department of Biostatistics and Health Informatics, Institute of Psychiatry, Psychology and Neuroscience, King's College London, London, UK; d Centre for Implementation Science, Department of Health Services and Population Research, Institute of Psychiatry, Psychology and Neuroscience, King's College London, London, UK; e Public Health England, London, UK; f Action on Smoking and Health UK, London, UK

**Keywords:** Smoking, Electronic cigarettes, E-cigarettes, Young people, Youth, Adolescent, Longitudinal studies, Nicotine, Tobacco

## Abstract

**Purpose:**

Electronic cigarette (e-cigarette) use is associated with smoking initiation among young people; however, it is also possible that smoking is associated with e-cigarette initiation. This study explores these associations among young people in Great Britain.

**Methods:**

A longitudinal survey of 1,152 11- to 18-year-olds was conducted with baseline in April 2016 and follow-up between August and October 2016. Logistic regression models and causal mediation analyses assessed whether (1) ever e-cigarette use and escalation were associated with smoking initiation (ever smoking at follow-up) among baseline never smokers (n = 923), and (2) ever smoking and escalation were associated with e-cigarette initiation (ever e-cigarette use at follow-up) among baseline never e-cigarette users (n = 1,020).

**Results:**

At baseline, 19.8% were ever smokers and 11.4% were ever e-cigarette users. Respondents who were ever e-cigarette users (vs. never users, 53% vs. 8%, odds ratio [OR] = 11.89, 95% confidence interval [CI] = 3.56–39.72) and escalated their e-cigarette use (vs. did not, 41% vs. 8%, OR = 7.89, 95% CI = 3.06–20.38) were more likely to initiate smoking. Respondents who were ever smokers (vs. never smokers, 32% vs. 4%, OR = 3.54, 95% CI = 1.68–7.45) and escalated their smoking (vs. did not, 34% vs. 6%, OR = 5.79, 95% CI = 2.55–13.15) were more likely to initiate e-cigarette use. There was a direct effect of ever e-cigarette use on smoking initiation (OR = 1.34, 95% CI = 1.05–1.72), and ever smoking on e-cigarette initiation (OR = 1.08, 95% CI = 1.01–1.17); e-cigarette and smoking escalation, respectively, did not mediate these effects.

**Conclusions:**

Among young people in Great Britain, ever e-cigarette use is associated with smoking initiation, and ever smoking is associated with e-cigarette initiation.

**Implications and Contribution**

This study employs a causal inference approach to provide further support for the association between ever e-cigarette use and smoking initiation, and additionally finds that ever smoking is associated with e-cigarette initiation, among young people.

Alt-text: Unlabelled box

There are an estimated 2.9 million current adult electronic cigarette (e-cigarette) users in Great Britain [1]. Concerns have been expressed about the impact of e-cigarette use on cigarette smoking, particularly among young people [2], [3], [4]. There is some evidence that trial of e-cigarettes among young people aged 11–18 years in Great Britain is rising (from 3.7% in 2013 to 9.3% in 2016) [5]. However, regular (at least monthly) use among young people is low, and increases in regular use are mainly restricted to current smokers (from 20.2% in 2015 to 27.2% in 2016), with regular use by never smokers remaining rare (.6% in 2015 to .4% in 2016) [5].

Cross-sectional studies have found that young people who use e-cigarettes are more likely to smoke [6], [7], intend to smoke [8], [9], and be susceptible to smoking [10] than those who do not. On the other hand, among young people in Great Britain, ex- and current smokers are more likely to intend to use e-cigarettes than never smokers [11]. It is therefore difficult to determine whether there is any causality, and it is likely that there is an underlying factor driving both smoking and e-cigarette use.

Several longitudinal studies of U.S. youth have found baseline e-cigarette use is associated with smoking initiation [12], [13], [14], [15], [16], [17], past six-month smoking [18], and past-month smoking [19] at follow-up. A meta-analysis of these studies has confirmed the strength and consistency of these associations [4], and the association between ever e-cigarette use and smoking initiation has since been replicated in England [20] and Scotland [21].

Although each of the above studies exploring the association between e-cigarette use and smoking control for a variety of factors associated with smoking, there remains the presence of extraneous variables, which may be related to both smoking and e-cigarette use. Furthermore, some researchers propose that certain psychosocial processes lead to vulnerability to any drug use [22], [23]. One study [18] explored whether the association between smoking and e-cigarettes works both ways, and found that not only was use of e-cigarettes at baseline associated with past six-month smoking at follow-up, but also smoking at baseline was associated with past six-month e-cigarette use at follow-up. Furthermore, among young people in Argentina, current smoking was associated with e-cigarette initiation one and a half years later [24].

Despite the above research, the relative contributions of e-cigarette use to smoking initiation, and smoking to e-cigarette initiation, have not been formally assessed. All studies in this field with the exception of Wills and colleagues [15] have relied on standard regression models [12], [13], [14], [16], [17], [18], [19], [20], [21], [24], which allow only limited conclusions to be drawn regarding the pathways between these products. Therefore, in this study, we have included causal mediation analyses [25] to investigate the causal influence of e-cigarette use on smoking initiation, and smoking on e-cigarette initiation.

This study is the first to our knowledge to explore the longitudinal association between (1) ever e-cigarette use and smoking initiation (ever smoking at follow-up) among baseline never smokers, and (2) ever smoking and e-cigarette initiation (ever e-cigarette use at follow-up) among baseline never e-cigarette users, among young people in Great Britain. We additionally explore whether escalation of each product between baseline and follow-up is associated with initiation of the alternative product, and employ causal mediation analyses for the identification of mediating factors [25] to investigate specific pathways between the two products.

## Methods

### Design

This study used data from the 2016 Action on Smoking and Health Great Britain Youth longitudinal survey. A non-probability quota sampling approach was adopted using Ipsos MORI's online panels to recruit respondents aged 11–18 years. Quotas were set in respect of age, gender, and Government Office Region (GOR) using data from Eurostat 2012 to ensure sample representativeness. Respondents were invited by email to participate in an online survey about smoking between April 6 and 20 with follow-up between August 5 and October 7, 2016. Up to eight email reminders were sent to maximize follow-up rates. Each wave took approximately 10 minutes to complete, and financial incentives were provided via a prize draw. Informed consent to take part in the surveys was provided either by the parents of those aged 11–15 years or by those individuals aged 16–18 years. Ethical approval for the analyses in this paper was not required as this study used secondary pre-existing data.

Ipsos MORI's online panel applicants consist of volunteers from the general public. These panel applicants are validated by a means of sophisticated vetting procedures using a variety of recruitment channels. Shortly after joining, panelists' survey-taking behavior is tested, with those most likely to make intentional or unintentional errors on future surveys deactivated. Subsequently, panelists' behavior is monitored and tracked across all surveys for quality reasons.

### Sample

**Figure 1:**
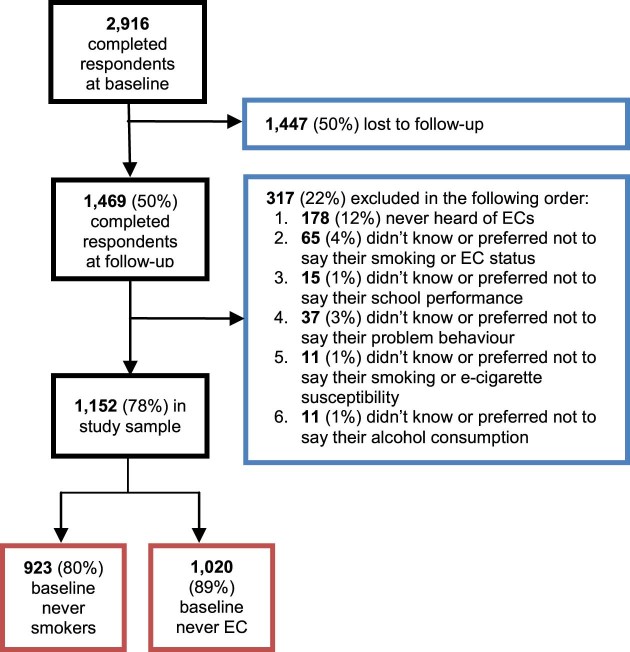
Flow diagram illustrating the respondent selection process. EC = electronic cigarette.

The baseline survey was completed by 2,916 respondents aged 11–18 years, of whom 1,469 (50%) successfully completed the follow-up survey. We excluded 317 respondents (22%) who had never heard of e-cigarettes and selected “don't know” or “prefer not to say” to some questions (see full breakdown in Figure 1). This left a final study sample of 1,152, of whom 923 (80%) were baseline never smokers and 1,020 (89%) were baseline never e-cigarette users (Figure 1).

### Measures

#### Smoking and e-cigarette status

At baseline, respondents were classified as never smokers (never smoked, not even a puff) or ever smokers; at follow-up, respondents were classified as never smokers or initiated smoking (never smokers at baseline but ever smokers at follow-up). At follow-up, respondents were further classified as having escalated smoking (increased their smoking between baseline and follow-up, e.g., escalating from never smoking to trying smoking, from smoking sometimes to smoking between one and six cigarettes a week) or not escalated smoking. Respondents were classified using the same procedure for e-cigarette use. Respondents who had never heard of e-cigarettes (n = 178), and those who responded with “Prefer not to say” or “Don't know” to the smoking or e-cigarette question at either baseline or follow-up (n = 65) were excluded from all analyses. Full item wording and response options are available in Table A1 (Supplementary Data).

#### Covariates (assessed at baseline only)

Age (11–13, 14–15, 16–18), gender (male, female), school performance (1–4, below average to excellent), problem behavior (2–8, 8 = greater problem behavior), monthly alcohol use (yes, no), smoking susceptibility (susceptible, not susceptible) [26], e-cigarette susceptibility (susceptible, not susceptible—to mirror smoking susceptibility [26]), some friends smoke (yes, no, not applicable/don't know), some friends use e-cigarettes (yes, no, not applicable/don't know), at least one parent smokes (yes, no), at least one parent uses e-cigarettes (yes, no), sibling(s) smoke (yes, no, not applicable/don't know), sibling(s) use e-cigarettes (yes, no, not applicable/don't know), public approve of smoking (yes, no), and public approve of e-cigarettes (yes, no) [27]. For school performance, problem behavior, monthly alcohol use, and smoking and e-cigarette susceptibility, “Don't know” and “Prefer not to say” responses were excluded from all analyses. Covariates specific to smoking were selected based on the previous literature [12], [15], [18], [26], [27], [28] and friend, parental, and sibling e-cigarette use and public approval of e-cigarettes were also included to mirror the similar smoking measures and to explore potential shared risk factors for each product. Full item wording, response options, and further details on coding for all covariates are available in Table A1 (Supplementary Data).

### Statistical analysis

We used unadjusted logistic regressions to compare respondents lost to follow-up with those retained and included in the study sample. We then used chi-square tests to compare smoking and e-cigarette status at baseline and follow-up. We used unadjusted and adjusted logistic regressions to explore the associations between (1) ever e-cigarette use at baseline and e-cigarette escalation between baseline and follow-up with smoking initiation at follow-up among baseline never smokers (n = 923), and (2) ever smoking at baseline and smoking escalation between baseline and follow-up with e-cigarette initiation at follow-up among baseline never e-cigarette users (n = 1,020). In adjusted models, we adjusted for all covariates described in the Measures section.

To decompose the causal effect of e-cigarette use on smoking initiation, and smoking on e-cigarette initiation, we used causal mediation analyses using the parametric g-computation procedure [25]. Mediation analyses go beyond standard regression models, which can estimate the associations between use of both products, by disentangling different pathways that could explain the effect of an exposure on an outcome. Furthermore, when a potential mediator is treated as confounder in standard regression models, spurious associations may arise. The most commonly used mediation analysis in epidemiology is based on the Baron and Kenny approach [29], in which the total effect of an exposure on an outcome, the effect of the exposure explained by a given set of mediators (indirect effect), and the effect of the exposure unexplained by those same mediators (direct effect) can be defined. This approach has four main problems as it (1) assumes no unmeasured confounding between mediator and outcome, (2) assumes no interactions between exposure and mediator on outcome, (3) does not extend to nonlinear models, and (4) assumes correctly specified models.

Causal mediation analysis has arisen from the causal inference literature [30] and addressed problems of the Baron and Kenny approach [29] under the potential outcomes framework, first by defining (using potential outcomes) precisely what is meant by direct and indirect effects, second by giving clear assumptions under which they can be identified, and third by generalizing the statistical methods available for carrying out such analyses to allow for nonlinearities, interactions, discrete outcomes, and semiparametric estimation [31]. We therefore use the parametric g-computation procedure under this framework as it can quantify reliable direct and indirect causal effects for binary variables, and produces narrow confidence intervals to allow for stronger conclusions to be made regarding observed associations [25], [32]. The g-computation procedure is discussed in detail elsewhere [25], [31], [32], but primarily relies on the parametric modeling assumptions shared with logistic regression and, to infer causality, assumes no unmeasured confounding. It has been applied to survey data previously [33].

**Figure 2:**
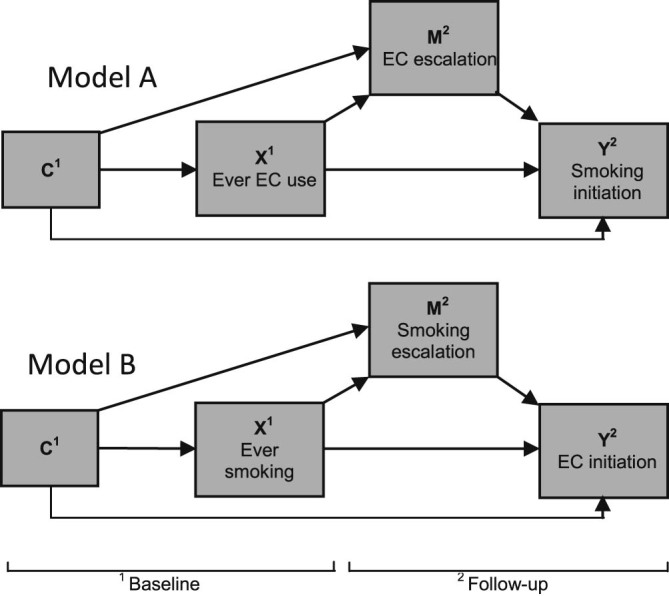
Conceptual causal diagrams for mediation and confounding. C = Covariate(s); X = Exposure; M = Mediator; Y = Outcome, EC = E-cigarette. Model A specifies baseline ever e-cigarette use as the exposure, e-cigarette escalation at follow-up as the mediator, and smoking initiation at follow-up as the outcome. Model B specifies baseline ever smoking as the exposure, smoking escalation at follow-up as the mediator, and e-cigarette initiation at follow-up as the outcome.

To assess the causal influence of e-cigarette use on smoking initiation, we specified a direct effect from ever e-cigarette use at baseline to smoking initiation at follow-up and an indirect effect acting through e-cigarette escalation between baseline and follow-up (mediator). We used the same approach to assess the causal influence of ever smoking on e-cigarette initiation at follow-up with smoking escalation between baseline and follow-up acting as a mediator. The causal diagrams for each model are shown in Figure 2. In the causal mediation analyses, all covariates described in the Measures section were specified as baseline confounders. The g-computation estimates were converted to odds ratios via exponentiation.

For attrition analysis and causal mediation analyses, we used unweighted data; for all other analyses, we used weighted data unless otherwise specified. Data were weighted according to age, gender, and GOR using data from the Eurostat 2012, and adjusted for attrition on age, gender, GOR, ever smoking, and ever e-cigarette use. Missing data were excluded listwise from all analyses (see Figure 1).

## Results

Table 1 shows the characteristics of the study sample at baseline (n = 1,152) compared with respondents lost to follow-up and who would have otherwise been excluded (because of not having heard of e-cigarettes or selecting “don't know” or “prefer not to say” on key variables and covariates) (n = 1,225). Respondents were more likely to be lost to follow-up if they had ever smoked and ever used an e-cigarette, and also differed on all covariates included in the study except smoking susceptibility and having at least one parent who uses e-cigarettes.

**Table 1:** Respondent characteristics of the study sample at baseline (n = 1,152) and comparison with those lost to follow-up who would have otherwise been excluded (n = 1,225)

	Study sample (n = 1,152)	Lost to follow-up and excluded (n = 1,225)	OR (95% CI)
Ever smoked	229 (19.88)	382 (31.18)	**.55 (.45–.66)**
Ever used e-cigarettes	132 (11.46)	297 (24.24)	**.40 (.32–.51)**
Female	620 (53.82)	564 (46.04)	**1.37 (1.16–1.61)**
Age			
11–13	438 (38.02)	375 (30.61)	
14–15	338 (29.34)	263 (21.47)	1.10 (.89–1.36)
16–18	376 (32.64)	587 (47.92)	**.55 (.45–.66)**
School performance (1–4, 4 = excellent), mean (SD)	3.05 (.8)	2.97 (.8)	**1.11 (1.01–1.22)**
Problem behavior (2–8, 8 = high), mean (SD)	2.93 (1.2)	3.30 (1.4)	**.80 (.75–.86)**
Monthly alcohol use	269 (23.35)	407 (33.22)	**.61 (.51–.73)**
Susceptible to smoking	146 (12.67)	151 (12.33)	.86 (.67–1.11)
Susceptible to using e-cigarettes	264 (22.92)	330 (26.94)	**.63 (.52–.77)**
Some friends smoke			
No	371 (32.2)	279 (22.78)	
Yes	727 (63.11)	894 (72.98)	**.61 (.51–.73)**
DK/NA	54 (4.69)	52 (4.24)	.78 (.52–1.18)
Some friends use e-cigarettes			
No	684 (59.38)	526 (42.94)	
Yes	399 (34.64)	620 (50.61)	**.49 (.42–.59)**
DK/NA	69 (5.99)	79 (6.45)	**.67 (.48–.95)**
At least one parent smokes	343 (29.77)	413 (33.71)	**.83 (.70–.99)**
At least one parent uses e-cigarettes	182 (15.8)	221 (18.04)	.85 (.69–1.06)
Sibling(s) smokes			
No	918 (79.69)	935 (76.33)	
Yes	127 (11.02)	191 (15.59)	**.68 (.53–.86)**
NA/DK	107 (9.29)	99 (8.08)	1.10 (.83–1.47)
Sibling(s) use e-cigarettes			
No	992 (86.11)	1016 (82.94)	
Yes	54 (4.69)	119 (9.71)	**.46 (.33–.65)**
NA/DK	106 (9.20)	90 (7.35)	1.21 (.90–1.62)
Public approve of smoking	33 (2.86)	62 (5.06)	**.55 (.36–.85)**
Public approve of e-cigarettes	43 (3.73)	90 (7.35)	**.49 (.34–.71)**

All data are unweighted. Significant associations (*p* < .05) are highlighted in **bold.**

N (%) of the samples are reported unless otherwise stated.

At baseline, 229 respondents (19.9%) had ever smoked (Table 1), and this increased to 301 (26.0%) at follow-up (χ^2^ = 834.32, *p* < .001). Of the 229 baseline ever smokers, 111 (48.5%) were also ever e-cigarette users; of the 923 baseline never smokers, 21 (2.3%) were ever e-cigarette users. At baseline, 132 respondents (11.5%) had ever used an e-cigarette (Table 1), increasing to 204 (17.6%) at follow-up (χ^2^ = 761.74, *p* < .001). Of the 132 baseline ever e-cigarette users, 111 (84.0%) were also ever smokers; of the 1,020 baseline never e-cigarette users, 118 (11.6%) were ever smokers. At baseline, only 56 (4.9%) respondents smoked monthly or more and 24 (2.1%) used an e-cigarette monthly or more.

Compared with baseline never e-cigarette users, ever e-cigarette users were more likely to initiate smoking at follow-up (Table 2). Furthermore, respondents who escalated e-cigarette use between baseline and follow-up were also more likely to initiate smoking at follow-up compared with those who did not (Table 2).

**Table 2:** Associations between smoking initiation at follow-up and e-cigarette use and all covariates, among baseline never smokers (n = 923)

	n (% initiated smoking)	Unadjusted		Adjusted model 1^a^		Adjusted model 2^b^	
		OR (95% CI)	*p*	OR (95% CI)	*p*	OR (95% CI)	*p*
Baseline EC use							
Never	902 (8.2)	1.00		1.00		1.00	
Ever	21 (52.6)	**12.41 (4.53–33.99)**	**<.001**	**10.57 (3.33–33.50)**	**<.001**	**11.89 (3.56–39.72)**	**<.001**
Follow-up EC use							
No escalation	882 (8.1)	1.00		—	—	1.00	
Escalation	41 (41.0)	**7.94 (3.75–16.82)**	**<.001**	—	—	**7.89 (3.06–20.38)**	**<.001**
Age							
11–13	397 (4.4)	1.00		1.00		1.00	
14–15	270 (6.3)	1.45 (.71–2.97)	.312	1.22 (.54–2.73)	.636	1.35 (.58–3.15)	.485
16–18	256 (16.1)	**4.12 (2.19–7.76)**	**<.001**	**4.02 (1.72–9.40)**	**.001**	**4.98 (2.07–12.00)**	**<.001**
Gender							
Male	428 (10.8)	1.00		1.00		1.00	
Female	495 (8.5)	.77 (.46–1.30)	.331	.90 (.48–1.68)	.738	.91 (.47–1.76)	.786
School perf. (1–4, 4 = excellent)^c^	2.93 (.9)	.76 (.53–1.08)	.124	.91 (.64–1.29)	.596	.90 (.64–1.29)	.579
Problem beh. (2–8, 8 = high)^c^	3.05 (1.3)	**1.31 (1.03–1.66)**	**.028**	1.06 (.82–1.37)	.659	1.05 (.81–1.36)	.705
Monthly alcohol use							
No	790 (7.8)	1.00		1.00		1.00	
Yes	133 (18.1)	**2.61 (1.42–4.80)**	**.002**	1.64 (.82–3.30)	.165	1.32 (.61–2.86)	.480
Smoking susceptibility							
No	777 (7.9)	1.00		1.00		1.00	
Yes	146 (19.8)	**2.88 (1.57–5.29)**	**.001**	**2.38 (1.17–4.84)**	**.016**	**2.61 (1.23–5.52)**	**.012**
Some friends smoke							
No	355 (5.4)	1.00		1.00		1.00	
Yes	515 (12.9)	**2.60 (1.34–5.07)**	**.005**	1.48 (.66–3.34)	.341	1.28 (.57–2.87)	.555
NA/DK	53 (1.9)	.35 (.04–2.76)	.317	.30 (.04–2.43)	.258	.29 (.04–2.36)	.246
Some friends use EC							
No	598 (8.6)	1.00		1.00		1.00	
Yes	264 (11.0)	1.32 (.73–2.40)	.358	**.47 (.24–.93)**	**.029**	**.35 (.17–.75)**	**.007**
NA/DK	61 (15.1)	1.90 (.73–4.94)	.188	1.99 (.78–5.10)	.150	1.80 (.72–4.51)	.212
At least one parent smokes							
No	676 (6.8)	1.00		1.00		1.00	
Yes	247 (18.0)	**2.99 (1.72–5.20)**	**<.001**	**2.97 (1.62–5.44)**	**<.001**	**2.65 (1.37–5.12)**	**.004**
At least one parent uses EC							
No	802 (8.4)	1.00		1.00		1.00	
Yes	121 (18.8)	**2.54 (1.35–4.76)**	**.004**	1.47 (.70–3.07)	.304	1.33 (.65–2.73)	.437
Sibling(s) smoke							
No	761 (8.5)	1.00		1.00		1.00	
Yes	71 (20.8)	**2.83 (1.23–6.51)**	**.015**	.75 (.30–1.84)	.527	.84 (.33–2.16)	.723
NA/DK	91 (10.4)	1.25 (.56–2.82)	.584	1.65 (.56–4.92)	.365	1.94 (.66–5.69)	.226
Sibling(s) use EC							
No	810 (9.3)	1.00		1.00		1.00	
Yes	28 (24.3)	**3.13 (1.09–9.01)**	**.034**	2.16 (.54–8.58)	.274	1.59 (.35–7.27)	.551
NA/DK	85 (9.3)	1.00 (.41–2.41)	.998	.72 (.20–2.53)	.604	.67 (.19–2.41)	.543
Public approve of smoking							
No	903 (9.5)	1.00		1.00		1.00	
Yes	20 (20.5)	2.45 (.60–9.96)	.209	1.33 (.34–5.16)	.676	1.87 (.48–7.19)	.365
Public approve of ECs							
No	907 (9.7)	1.00		1.00		1.00	
Yes	16 (9.8)	1.00 (.20–4.99)	.997	.39 (.07–2.05)	.263	.40 (.08–1.92)	.252

Adjusted model 1 constant OR = .02 (95% CI = .00–.11) *p* < .001. Adjusted model 2 constant OR = .02 (95% CI = .00–.10), *p* < .001. N and % illustrate the number and percentage of individuals who initiated smoking at follow-up. All n use unweighted data, % and analyses use weighted data.

Significant associations (*p* < .05) are highlighted in **bold.**

beh. = behavior; EC = e-cigarette; perf. = performance.

a Adjusted model 1 is adjusted for all variables listed except follow-up EC use.

b Adjusted model 2 is adjusted for all variables listed.

c Mean(SD) reported, mean (SD) for never smoked at follow-up: school performance = 3.12 (.8), problem behavior = 2.71 (1.0).

Compared with baseline never smokers, ever smokers were more likely to initiate e-cigarette use at follow-up (Table 3). Furthermore, respondents who escalated smoking between baseline and follow-up were also more likely to initiate e-cigarette use at follow-up compared with those who did not (Table 3).

**Table 3:** Associations between e-cigarette initiation at follow-up and smoking and all covariates, among baseline never e-cigarette users (n = 1,020)

	n (% initiated EC use)	Unadjusted		Adjusted model 1^a^		Adjusted model 2^b^	
		OR (95% CI)	*p*	OR (95% CI)	*p*	OR (95% CI)	*p*
Baseline smoking							
Never	902 (4.1)	1.00		1.00		1.00	
Ever	118 (32.4)	**9.48 (5.36–16.76)**	**<.001**	**3.69 (1.88–7.23)**	**<.001**	**3.54 (1.68–7.45)**	**.001**
Follow-up smoking							
No escalation	932 (5.9)	1.00		—	—	1.00	
Escalation	88 (33.5)	**8.00 (4.36–14.69)**	**<.001**	—	—	**5.79 (2.55–13.15)**	**<.001**
Age							
11–13	413 (5.6)	1.00		1.00		1.00	
14–15	294 (6.1)	1.11 (.54–2.27)	.779	.65 (.29–1.43)	.285	.57 (.25–1.27)	.168
16–18	313 (12.5)	**2.41 (1.29–4.51)**	**.006**	.69 (.31–1.55)	.374	.48 (.19–1.18)	.109
Gender							
Male	468 (10.2)	1.00		1.00		1.00	
Female	552 (7.3)	.70 (.41–1.17)	.171	.77 (.41–1.43)	.404	.73 (.39–1.37)	.331
School perf. (1–4, 4 = excellent)^c^	2.67 (.9)	**.57 (.42–.78)**	**<.001**	.81 (.58–1.14)	.226	.79 (.55–1.12)	.183
Problem beh. (2–8, 8 = high)^c^	3.51 (1.4)	**1.62 (1.30–2.03)**	**<.001**	1.20 (.93–1.53)	.154	1.13 (.87–1.47)	.352
Monthly alcohol use							
No	824 (5.0)	1.00		1.00		1.00	
Yes	196 (20.6)	**4.93 (2.87–8.47)**	**<.001**	**2.66 (1.27–5.61)**	**.010**	**2.40 (1.08–5.33)**	**.032**
EC susceptibility							
No	756 (5.1)	1.00		1.00		1.00	
Yes	264 (18.9)	**4.39 (2.51–7.67)**	**<.001**	1.53 (.83–2.83)	.173	1.67 (.86–3.27)	.131
Some friends smoke							
No	363 (2.4)	1.00		1.00		1.00	
Yes	603 (12.3)	**5.58 (2.44–12.73)**	**<.001**	1.97 (.86–4.50)	.107	1.95 (.87–4.36)	.105
NA/DK	54 (5.5)	2.34 (.56–9.84)	.247	3.24 (.60–17.36)	.170	4.31 (.88–21.13)	.071
Some friends use EC							
No	660 (5.7)	1.00		1.00		1.00	
Yes	293 (15.9)	**3.14 (1.81–5.45)**	**<.001**	**2.69 (1.48–4.87)**	**.001**	**3.03 (1.63–5.64)**	**<.001**
NA/DK	67 (6.4)	1.15 (.31–4.19)	.835	1.10 (.20–6.14)	.915	.78 (.14–4.54)	.785
At least one parent smokes							
No	733 (6.6)	1.00		1.00		1.00	
Yes	287 (14.9)	**2.47 (1.45–4.23)**	**.001**	1.88 (.91–3.91)	.090	1.45 (.61–3.46)	.405
At least one parent uses EC							
No	884 (7.6)	1.00		1.00		1.00	
Yes	136 (17.3)	**2.54 (1.38–4.67)**	**.003**	2.34 (1.00–5.47)	.051	2.1 (.87–5.07)	.097
Sibling(s) smoke							
No	830 (7.4)	1.00		1.00		1.00	
Yes	94 (24.0)	**3.94 (2.00–7.75)**	**<.001**	1.49 (.66–3.36)	.332	1.64 (.69–3.91)	.266
NA/DK	96 (3.9)	.51 (.16–1.61)	.251	.36 (.06–2.11)	.258	.27 (.04–1.93)	.193
Sibling(s) use EC							
No	899 (8.3)	1.00		1.00		1.00	
Yes	31 (29.9)	**4.69 (1.50–14.66)**	**.008**	1.46 (.39–5.43)	.576	.92 (.28–3.09)	.895
NA/DK	90 (5.6)	.66 (.23–1.83)	.420	1.03 (.21–5.11)	.969	1.10 (.19–6.27)	.917
Public approve of smoking							
No	1000 (9.0)	1.00		1.00		1.00	
Yes	20 (2.8)	.29 (.04–2.22)	.233	**.09 (.01–.88)**	**.038**	.15 (.02–1.22)	.076
Public approve of ECs							
No	995 (8.5)	1.00		1.00		1.00	
Yes	25 (20.9)	2.84 (.95–8.50)	.061	.99 (.31–3.15)	.987	1.32 (.34–5.15)	.689

Adjusted model 1 constant OR = .02 (95% CI = .00–.07) *p* < .001. Adjusted model 2 constant OR = .02 (95% CI = .00–.10), *p* < .001. N and % illustrate the number and percentage of individuals who initiated EC use at follow-up. All n use unweighted data, % and analyses use weighted data.

Significant associations (*p* < .05) are highlighted in **bold.**

beh. = behavior; EC = e-cigarette; perf. = performance.

a Adjusted model 1 is adjusted for all variables listed except follow-up smoking.

b Adjusted model 2 is adjusted for all variables listed.

c Mean (SD) reported, mean (SD) for never used EC at follow-up: school performance = 3.08 (.8), problem behavior = 2.77 (1.0).

Having some friends who use an e-cigarette reduced the likelihood of smoking initiation (Table 2) but increased the likelihood of e-cigarette initiation (Table 3). Being older, susceptible to smoking, and having at least one parent who smokes were associated with an increased likelihood of smoking initiation (Table 2). Monthly alcohol use and no perceived public approval of smoking were associated with an increased likelihood of e-cigarette initiation (Table 3).

In the causal mediation analysis (Figure 2, model A), baseline ever e-cigarette use had a direct causal effect on smoking initiation at follow-up (odds ratio [OR] = 1.34, 95% confidence interval [CI] = 1.05–1.72, *p* = .018), and there was a significant total causal effect of the model (OR = 1.35, 95% CI = 1.04–1.74, *p* = .022). However, there was no indirect effect of baseline ever e-cigarette use on smoking initiation at follow-up mediated by e-cigarette escalation between baseline and follow-up (OR = 1.00, 95% CI = .91–1.11, *p* = .983).

In the causal mediation analysis (Figure 2, model B), baseline ever smoking had a direct causal effect on e-cigarette initiation at follow-up (OR = 1.08, 95% CI = 1.01–1.17, *p* = .034), and there was a significant total causal effect of the model (OR = 1.11, 95% CI = 1.03–1.20, *p* = .006). However, there was no indirect effect of baseline ever smoking on e-cigarette initiation at follow-up mediated by smoking escalation between baseline and follow-up (OR = 1.03, 95% CI = .99–1.06, *p* = .106).

## Discussion

This study was the first to explore the longitudinal association between e-cigarette use and smoking initiation, and smoking and e-cigarette initiation among young people in Great Britain, and to assess the relative contribution of these associations using a causal inference approach. In the logistic regression analyses, we found evidence for a prospective association between ever e-cigarette use and smoking initiation, and between ever smoking and e-cigarette initiation. We also found that escalation of each product (e-cigarettes and smoking) between baseline and follow-up was associated with initiation of the alternative product. The causal mediation analyses confirmed the direct effect of baseline ever e-cigarette use on smoking initiation, and baseline ever smoking on e-cigarette initiation, but found that e-cigarette and smoking escalation, respectively, did not mediate these effects.

This study provides insight into the impact of e-cigarette use on smoking and vice versa in young people; however, the findings must be considered in the light of some limitations. Attrition was high and respondents lost to follow-up differed substantially from those retained, potentially reducing generalizability to ever smokers, ever e-cigarette users, males, older respondents, and those with poorer school performance and greater problem behavior.

Although this study controlled for a variety of factors previously associated with smoking and e-cigarette use to enhance approximation of the models, there are still several factors that were not included that may contribute to the observed association between these products [28]. Examples may include curiosity, sensation seeking, liking, or disliking the effects of smoking/e-cigarettes, expectancies of smoking/e-cigarettes, mental ill health, and use of other drugs [28]. Furthermore, there are likely to be contributing factors that cannot be easily measured in surveys such as biological or genetic vulnerabilities, although drug use and parent's smoking and e-cigarette use may act as an indicator of these. Larger sample sizes are required to enable this substantial number of covariates to be assessed and meaningfully interpreted.

Another important limitation is that this study uses the outcomes smoking initiation and e-cigarette initiation defined as progressing from never to ever use of each product. This is similar to some previous studies [12], [13], [14], [15], [16], [21], [24], yet the use of such broad measures has been criticized for providing limited evidence of progression to any significant smoking behavior [28], [34]. However, because of low prevalence rates of monthly or more smoking (5%) and e-cigarette use (2%) in this study's sample, options for refining the measures were limited. Therefore, although the present study found an association between ever smoking and ever e-cigarette use, these cannot be generalized to current or regular use, and it cannot be determined whether e-cigarette experimentation leads to regular smoking. Such questions are critical in this area of research. Surveys with multiple waves across several years with larger sample sizes are needed to enable higher numbers of ever and current smokers and e-cigarette users, and further dissect the association between the two products.

Despite the above limitations, this study has several strengths. It was the first to explicitly explore the association not only between e-cigarette use at baseline and smoking initiation at follow-up but additionally smoking at baseline and e-cigarette initiation at follow-up. Moreover, a novel statistical approach (causal mediation analysis [25]) was used to explore whether the association between baseline ever e-cigarette use and smoking initiation at follow-up was mediated by escalation of e-cigarette use between survey waves; the same procedure was also used to explore further the association between smoking and e-cigarette initiation. To our knowledge this has not been done previously. Finally, the sample was drawn from the general population in Great Britain using a quota sampling approach to enhance representativeness.

The rate of ever smoking in this study was 19.9% at baseline, which is lower than other findings in Great Britain in 2016 [5], but could be because of those lost at follow-up being more likely to smoke. The rate of ever e-cigarette use (11.5% at baseline) and findings that ever e-cigarette use was largely confined to those who had ever smoked, with a low proportion of never smokers having ever used e-cigarettes, was consistent with other findings in Great Britain [5], [35]. Furthermore, only 4% of never smokers initiated e-cigarette use (vs. 32% of ever smokers). This suggests that e-cigarettes are attracting few who have never smoked. Furthermore, monthly or more smoking and e-cigarette use was low, at 5% and 2%, respectively.

In the logistic regression analyses, e-cigarette escalation between baseline and follow-up was associated with smoking initiation, even when controlling for ever e-cigarette use; likewise, smoking escalation was associated with e-cigarette initiation when controlling for ever smoking. This represents a novel contribution to the literature, and further suggests the need for multi-wave surveys to explore dynamic changes in use of both products over time. Despite this, the causal mediation analyses, which as discussed allow for stronger conclusions to be made regarding observed observations, suggest that it is primarily ever use of that product that contributes to initiation of the alternative product.

Our findings are consistent with previous studies that found a prospective association between e-cigarette use at baseline and smoking at follow-up [4], [12], [13], [14], [15], [16], [17], [18], [19], [20], [21], and also with those who found a prospective association between smoking at baseline and e-cigarette use at follow-up [18], [24]. There are several possible reasons for the strong and reliable association between e-cigarettes and smoking in young people [18], [28], [36]. One interpretation is that e-cigarettes act as a “gateway” to smoking [3], [37]; however, this has been contested [28], [36], and our findings suggest that the association between e-cigarette initiation and smoking initiation may work both ways. Certain psychological processes (“common liabilities”) may lead to vulnerability of any drug use [22], [23]. Specifically, young people who exhibit curiosity, rebelliousness, and sensation-seeking may be more likely to experiment with both smoking and e-cigarettes. Future research should explore potential common liabilities pertaining to experimentation of both products, some of which were included in this study and others are proposed above.

Despite potential common liabilities and our findings that e-cigarette use is associated with smoking and vice versa, there are several important differences to consider between these products and the contexts in which they may be used. Among young people, e-cigarettes, compared with conventional cigarettes, have been described as more accessible and convenient [38], [39], have a greater capacity for continual novelty in terms of flavors and devices [39], and are perceived as less harmful in the UK [5], [39]. On the contrary, smoking is highly stigmatized in some societal groups [40]. Indeed, some have reported that e-cigarettes appeal to those who do not want to smoke but want to try the experience of “smoking” [38], [39].

Interestingly, friend's e-cigarette use increased the likelihood of e-cigarette initiation but reduced the likelihood of smoking initiation in adjusted models. This first association is unsurprising given the important role of peer influence on behavior. However, the protective effect of friend's e-cigarette use on smoking initiation warrants further investigation.

In conclusion, this study provides further support for the association between ever e-cigarette use and smoking initiation, and additionally finds that ever smoking is associated with e-cigarette initiation, among young people. Better understanding of these associations will aid policy makers with their efforts to develop an appropriate regulatory framework for both tobacco products and e-cigarettes.

## Funding Sources

This work was funded by Cancer Research UK grant code A21559. Thanks are also given to the UK Public Health Research Consortium (grant number PHPEHF50/13) for funding the development of some of the covariates included in this study.

## Supplementary Data

Table A1 Description of smoking status, e-cigarette status, and demographic and psychosocial covariates. Italics indicate notes that were not included in the item wording. Table A1
