# Supplementary material for: The Association Between Smoking and Electronic Cigarette Use in a Cohort of Young People
Source: J Adolesc Health. 2018 May;62(5):539–47. doi: 10.1016/j.jadohealth.2017.11.301 (PMC5938086; doi:10.1016/j.jadohealth.2017.11.301)
Supplement: Table A1 — Description of smoking status, e-cigarette status, and demographic and psychosocial covariates. Italics indicate notes that were not included in the item wording. [file mmc1.docx]

**Appendix**

| **Table A1.** Description of smoking status, e-cigarette status, and demographic and psychosocial covariates. Italics indicate notes that were not included in the item wording. | | |
| --- | --- | --- |
| **Variable** | **Item(s) and response options** | **Coding** |
| **Smoking status** | Which ONE of the following BEST applies to you?   1. I have never smoked cigarettes, not even a puff or two 2. I have only ever tried smoking cigarettes once 3. I have tried smoking cigarettes more than once but only a few times 4. I used to smoke sometimes but I never smoke cigarettes now 5. I sometimes smoke cigarettes now but less than once a month 6. I usually smoke cigarettes at least once a month but less than once a week *(due to an error in the survey this was only assessed at W2)* 7. I usually smoke between one and six cigarettes a week 8. I usually smoke more than six cigarettes a week 9. Prefer not to say | **Never smoker** (1)  **Ever smoker** (2-8)  **Smoking escalation**  (baseline: 1 and follow-up: 2-8, or baseline: 2 and follow-up: 3, or baseline: 2 and follow-up: 5-8, or baseline: 3 and follow-up: 5-8, or baseline: 4 and follow-up: 5-8, or baseline: 5 and follow-up: 6-8, or baseline: 7 and follow-up: 8)  **No smoking escalation** all other combinations  Excluded = 9 |
| **E-cigarette status** | (a) Have you ever heard of e-cigarettes? They are also sometimes called shisha pens, vaporisers or electronic cigarettes.   1. Yes, I have 2. No, I haven’t 3. Don’t know   *[Those who responded “Yes, I have” to the above were then asked:]*  (b) Which ONE of the following is closest to describing your experience of e-cigarettes?   1. I have never used an e-cigarette 2. I have only used an e-cigarette once 3. I have used an e-cigarette more than once but only a few times 4. I used to use e-cigarettes but I do not use e-cigarettes now 5. I use e-cigarettes sometimes, but no more than once a month 6. I use e-cigarettes more than once a month, but less than once a week 7. I use e-cigarettes more than once a week but not every day 8. I use e-cigarettes every day 9. Prefer not to say 10. Don’t know | **Never user** ((a) 1 and (b) 1)  **Ever user** ((a) 1 and (b) 2-8)  **E-cigarette escalation** (a) 1 and (b) baseline: 1 and follow-up: 2-8, or baseline: 2 and follow-up: 3, or baseline: 2 and follow-up: 5-8, or baseline: 3 and follow-up: 5-8, or baseline: 4 and follow-up: 5-8, or baseline: 5 and follow-up: 6-8, or baseline: 6 and follow-up: 7-8 or baseline: 7 and follow-up: 8)  **No e-cigarette escalation** all other combinations  Excluded = (a) 2, 3 or (b) 9, 10 |
| **Age** | (a) *Adult initially answering survey:* And which of these age groups do you fall into?   1. Under 16 2. 16-18 3. 19-24 4. 25-34 5. 35-44 6. 45-54 7. 55-56 8. Over 65   (b) *If (a) 3-8:* Which, if any, of the following apply to you? PLEASE SELECT ALL THAT APPLY. I am the parent or legal guardian of a child / children aged …   1. 11-13 that live(s) with me 2. 14-15 that live(s) with me 3. None of the above 4. Prefer not to say | **11-13** ((a) 3-8 and (b) 1)  **14-15** ((a) 3-8 and (b) 2)  **16-18** ((a) 2)  Excluded = (a) 1 or (b) 3-4 |
| **Gender** | Are you...   1. Male 2. Female | **Male** (1)  **Female** (2) |
| Continued below |  |  |
| **Table A1.** Description of smoking status, e-cigarette status, and demographic and psychosocial covariates. Italics indicate notes that were not included in the item wording (continued). | | |
| **Variable** | **Item(s) and response options** | **Coding** |
| **School performance** | How would you describe your grades last year, or in the last year of school that you attended?   1. Excellent 2. Good 3. Average 4. Below average 5. Don’t know 6. Prefer not to say | **Continuous between 1 and 4,** recoded so that 1 = below average, 4 = excellent  Excluded = 5-6 |
| **Problem behaviour** | (a) I get in trouble in school/I used to get in trouble at school.   1. Not at all like me 2. A little like me 3. Pretty much like me 4. Exactly like me 5. Don’t know 6. Prefer not to say   (b) I do things my parent(s) (or carer(s)) wouldn’t want me to do.   1. Not at all like me 2. A little like me 3. Pretty much like me 4. Exactly like me 5. Don’t know 6. Prefer not to say | **Added the scales to form a continuous scale between 2-8**, with 8 = greater problem behaviour  Excluded = 5-6 to (a) or (b) |
| **Monthly alcohol use** | How often do you have an alcoholic drink, if at all?   1. I never drink alcoholic drinks 2. Every day or almost every day 3. About twice a week 4. About once a week 5. About once a fortnight 6. About once a month 7. Only a few times a year 8. About once a year 9. Don’t know 10. Prefer not to say | **Yes** (2-6)  **No** (1,7-8)  Excluded = 9-10 |
| **Smoking susceptibility** | (a) If one of your friends offered you a tobacco cigarette, would you try it?   1. Definitely yes 2. Probably yes 3. Probably no 4. Definitely no 5. Prefer not to say   (b) Do you think that you will try a cigarette sometime in the next year?   1. Definitely yes 2. Probably yes 3. Probably no 4. Definitely no 5. Prefer not to say | **Not susceptible** (4 to (a) and (b))  **Susceptible** (1-3 to (a) or (b))  Excluded = 5 to (a) or (b) |
| Continued below |  |  |
|  |  |  |
| **Table A1.** Description of smoking status, e-cigarette status, and demographic and psychosocial covariates. Italics indicate notes that were not included in the item wording (continued). | | |
| **Variable** | **Item(s) and response options** | **Coding** |
| **E-cigarette susceptibility** | (a) If one of your friends offered you an e-cigarette, would you try it?   1. Definitely yes 2. Probably yes 3. Probably no 4. Definitely no 5. Prefer not to say   (b) Do you think that you will try an e-cigarette sometime in the next year?   1. Definitely yes 2. Probably yes 3. Probably no 4. Definitely no 5. Prefer not to say | **Not susceptible** (4 to (a) and (b))  **Susceptible** (1-3 to (a) or (b))  Excluded = 5 to (a) or (b) |
| **Some friends smoke** | Do any of these people that you know smoke tobacco cigarettes?  (a) Some friends of my own age   1. Yes 2. No 3. Not applicable 4. Don’t know   (b) Some friends older than me.   1. Yes 2. No 3. Not applicable 4. Don’t know   (c) Some friends younger than me.   1. Yes 2. No 3. Not applicable 4. Don’t know | **Yes** (1 to either (a), (b) or (c))  **No** (2 to (a), (b) and (c) OR 2 for either (a), (b) or (c) but 3 for the rest)  **Not applicable/don’t know** was included as a separate response option due to the large number who selected other combinations (3 or 4 for (a), (b) and (c)) |
| **Some friends use e-cigarettes** | Do any of these people that you know use e-cigarettes?  (a) Some friends of my own age.   1. Yes 2. No 3. Not applicable 4. Don’t know   (b) Some friends older than me.   1. Yes 2. No 3. Not applicable 4. Don’t know   (c) Some friends younger than me.   1. Yes 2. No 3. Not applicable 4. Don’t know | **Yes** (1 to either (a), (b) or (c))  **No** (2 to (a), (b) and (c) OR 2 for either (a), (b) or (c) but 3 for the rest)  **Not applicable/don’t know** was included as a separate response option due to the large number who selected other combinations (3 or 4 for (a), (b) and (c)) |
| Continued below | | |
| **Table A1.** Description of smoking status, e-cigarette status, and demographic and psychosocial covariates. Italics indicate notes that were not included in the item wording (continued). | | |
| **Variable** | **Item(s) and response options** | **Coding** |
| **At least one parent smokes** | Do any of these people that you know smoke tobacco cigarettes?  (a) Mother (or female carer)   1. Yes 2. No 3. Not applicable 4. Don’t know   (b) Father (or male carer)   1. Yes 2. No 3. Not applicable 4. Don’t know | **Yes** (1 to either (a) or (b))  **No** (all other response combinations, due to the small number of respondents who selected 3 and 4) |
| **At least one parent uses e-cigarettes** | Do any of these people that you know use e-cigarettes?  (a) Mother (or female carer)   1. Yes 2. No 3. Not applicable 4. Don’t know   (b) Father (or male carer)   1. Yes 2. No 3. Not applicable 4. Don’t know | **Yes** (1 to either (a) or (b))  **No** (all other response combinations, due to the small number of respondents who selected 3 and 4) |
| **Sibling(s) smoke** | Do any of these people that you know smoke tobacco cigarettes? Brother or sister.   1. Yes 2. No 3. Not applicable 4. Don’t know | **Yes** (1)  **No** (2)  **Not applicable/don’t know** was included as a separate response option due to the large number who selected other combinations (3 or 4) |
| **Sibling(s) use e-cigarettes** | Do any of these people that you know use e-cigarettes? Brother or sister.   1. Yes 2. No 3. Not applicable 4. Don’t know | **Yes** (1)  **No** (2)  **Not applicable/don’t know** was included as a separate response option due to the large number who selected other combinations (3 or 4) |
| **Public approval of smoking** | In your opinion, do the general public approve or disapprove of people your age smoking tobacco cigarettes?   1. Strongly approve 2. Approve 3. Neither approve nor disapprove 4. Disapprove 5. Strongly disapprove 6. Don’t know | **Yes** (1-2)  **No** (3-6) |
| **Public approval of e-cigarettes** | In your opinion, do the general public approve or disapprove of people your age using e- cigarettes?   1. Strongly approve 2. Approve 3. Neither approve nor disapprove 4. Disapprove 5. Strongly disapprove 6. Don’t know | **Yes** (1-2)  **No** (3-6) |
